# Pathways to multidrug-resistant tuberculosis diagnosis and treatment initiation: a qualitative comparison of patients’ experiences in the era of rapid molecular diagnostic tests

**DOI:** 10.1186/s12913-015-1145-0

**Published:** 2015-10-28

**Authors:** Pren Naidoo, Margaret van Niekerk, Elizabeth du Toit, Nulda Beyers, Natalie Leon

**Affiliations:** Desmond Tutu TB Centre, Department of Paediatrics and Child Health, Faculty of Medicine and Health Sciences, Stellenbosch University, Stellenbosch, South Africa; Health Systems Research Unit, South African Medical Research Council, Cape Town, South Africa

**Keywords:** GenoType MTBDRplus line probe assay, Xpert® MTB/RIF, Pathways to care, Health-seeking behaviour, Diagnostic and treatment delay, Obstacles to care

## Abstract

**Background:**

Although new molecular diagnostic tests such as GenoType MTBDR*plus* and Xpert® MTB/RIF have reduced multidrug-resistant tuberculosis (MDR-TB) treatment initiation times, patients’ experiences of diagnosis and treatment initiation are not known. This study aimed to explore and compare MDR-TB patients’ experiences of their diagnostic and treatment initiation pathway in GenoType MTBDR*plus* and Xpert® MTB/RIF-based diagnostic algorithms.

**Methods:**

The study was undertaken in Cape Town, South Africa where primary health-care services provided free TB diagnosis and treatment. A smear, culture and GenoType MTBDR*plus* diagnostic algorithm was used in 2010, with Xpert® MTB/RIF phased in from 2011–2013. Participants diagnosed in each algorithm at four facilities were purposively sampled, stratifying by age, gender and MDR-TB risk profiles. We conducted in-depth qualitative interviews using a semi-structured interview guide. Through constant comparative analysis we induced common and divergent themes related to symptom recognition, health-care access, testing for MDR-TB and treatment initiation within and between groups. Data were triangulated with clinical information and health visit data from a structured questionnaire.

**Results:**

We identified both enablers and barriers to early MDR-TB diagnosis and treatment. Half the patients had previously been treated for TB; most recognised recurring symptoms and reported early health-seeking. Those who attributed symptoms to other causes delayed health-seeking. Perceptions of poor public sector services were prevalent and may have contributed both to deferred health-seeking and to patient’s use of the private sector, contributing to delays. However, once on treatment, most patients expressed satisfaction with public sector care. Two patients in the Xpert® MTB/RIF-based algorithm exemplified its potential to reduce delays, commencing MDR-TB treatment within a week of their first health contact. However, most patients in both algorithms experienced substantial delays. Avoidable health system delays resulted from providers not testing for TB at initial health contact, non-adherence to testing algorithms, results not being available and failure to promptly recall patients with positive results.

**Conclusion:**

Whilst the introduction of rapid tests such as Xpert® MTB/RIF can expedite MDR-TB diagnosis and treatment initiation, the full benefits are unlikely to be realised without reducing delays in health-seeking and addressing the structural barriers present in the health-care system.

## Background

The World Health Organisation (WHO) identified the need to address multi-drug resistant tuberculosis (MDR-TB) as a public health crisis as one of five key priorities [1]. Improving MDR-TB control requires rapid, accurate diagnostics to enable early and appropriate treatment [1, 2] with benefit to both patients and the public through reductions in morbidity, mortality and transmission within communities [2, 3]. Advances have been made in the development, evaluation and routine use of rapid, accurate molecular diagnostic tests for MDR-TB. South Africa was an early adopter of two WHO approved tests, GenoType MTBDR*plus* (Hain Lifescience GmbH, Nehren, Germany) line probe assay (LPA) [4] and Xpert® MTB/RIF (Cepheid, Sunnyvale, CA, USA) (Xpert) [5].

Studies have shown that both LPA [6, 7] and Xpert [8] reduced MDR-TB diagnostic and treatment initiation times in comparison to previous tests. Test performance, as well as both patient and health system factors influence the potential of rapid diagnostics to reduce diagnostic and treatment delays [9]. Although not specific to MDR-TB, studies have found a number of factors associated with patient delay in accessing care in sub-Saharan Africa [10]. Fear of a positive human immunodeficiency virus (HIV) test or the stigma associated with this [11–13], the belief that symptoms, like cough, would resolve spontaneously or improve with self-medication [11, 13, 14], uncertainty about the cause of their illness and visits to multiple providers [13–15] all contributed to delays in seeking care. Health provider delays were influenced by the availability of laboratory resources, initial screening efficacy, timely and correct request for laboratory investigations and the coordination of patient management between different health-care providers [13, 16, 17].

There is a paucity of literature detailing MDR-TB patients’ experience of diagnosis and treatment initiation. This study aimed to explore and compare MDR-TB patients’ experiences of their pathway to diagnosis and treatment initiation in LPA and Xpert-based diagnostic algorithms. The study was part of a broader PROVE IT (Policy Relevant Outcomes from Validating Evidence on ImpacT) (http://www.treattb.org) evaluation that assessed the impact of new molecular tests on the diagnosis and treatment of TB in routine operational conditions. Impact analysis was guided by the Impact Assessment Framework [18] which provides a systematic, comprehensive approach to generating evidence to support decision-making for new diagnostics.

## Methods

### Study setting

The study took place in Cape Town, South Africa. Cape Town has a high TB burden with 28,658 TB cases and 953 MDR-TB cases notified in 2011 and a TB case notification rate of 752/100,000 population. Free TB diagnostic services were provided at 142 primary health-care (PHC) facilities offering two different service platforms. Community Health Centres (CHC) were large, busy facilities treating acutely ill adults and most offered only TB diagnostic services. Clinics tended to be smaller, focused on disease prevention and offered both TB diagnostic and treatment services. All TB tests were done at a central laboratory that received daily specimens via courier and recorded results in a networked, electronic laboratory database. Rapid, on-site HIV-testing was routinely offered to presumptive TB cases.

**Fig. 1:**
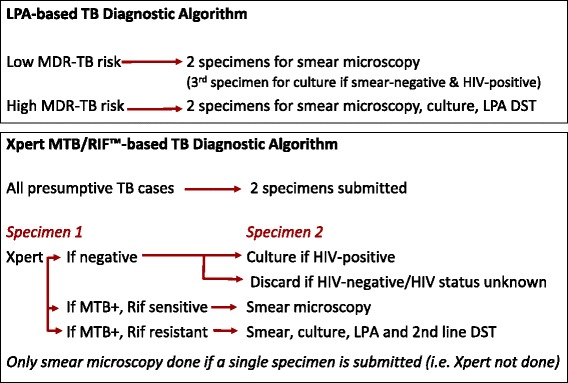
Testing in the LPA and Xpert-based TB diagnostic algorithms. High MDR-TB-risk presumptive cases refer to those with previous TB, an MDR-TB contact or from a congregate setting. In the LPA-based algorithm, only these cases were initially screened for drug susceptibility. Low MDR-risk presumptive TB cases would only be identified when 1^st^-line TB treatment regimens failed. In the Xpert-based algorithm in comparsion, all presumptive TB cases were simultaneously screened for TB and rifampicn resistance using Xpert

In 2010, a smear, culture and LPA-based diagnostic algorithm was used (Fig. 1) with LPA done on culture isolates or clinical specimens of *high* MDR-TB-risk presumptive cases (those with previous TB, an MDR-TB contact or from a congregate setting). Doctors at the TB-hospital reviewed case records and prescribed MDR-TB treatment but patients could initiate treatment at PHC facilities.

From 2011–2013, Xpert was phased in, replacing smear microscopy for *all* presumptive TB cases (Fig. 1). Full decentralisation of treatment occurred from 2012 with doctors at PHC facilities initiating standardised MDR-TB treatment without the need for prior case review at the TB-hospital.

### Sampling

**Fig. 2:**
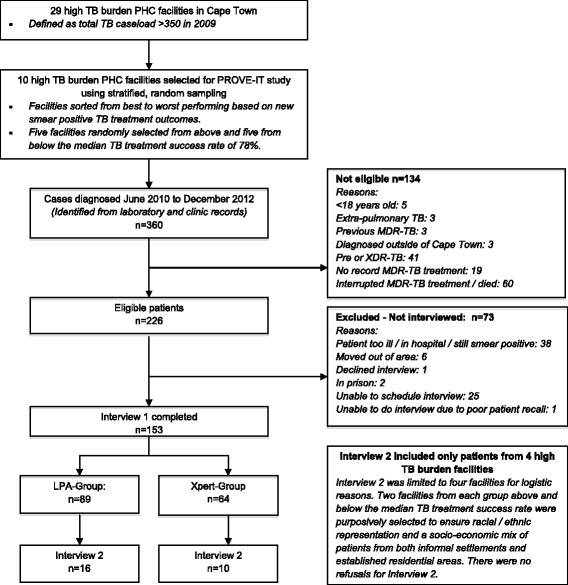
Primary health facility and MDR-TB patient selection. Patients in this study were part of a PROVE-IT observational cohort in 10 high TB-burden PHC facilities selected from a total of 29 that met the criteria of a high TB caseload in 2009. The flowchart indicates the selection of health facilities and patients for this study. Data from Interview 1 elicited quantitative information related to duration of illness, health-seeking visits and providers, cost incurred and socio-economic status data (used for costing purposes, to evaluate delay and to supplement information on patient pathways). Interview 2 was an in-depth qualitative interview exploring patients’ perspectives of their pathways to care and formed the basis for this manuscript

Patients in this study were part of a PROVE-IT observational cohort in 10 high TB-burden PHC facilities selected from a total of 29 that met the criteria of a TB caseload of >350 in 2009. Health facility and patient sampling details are provided in Fig. 2. We limited the number of facilities in this study for logistic reasons and purposively selected four of these facilities to ensure racial / ethnic representation and a socio-economic mix of patients from both informal settlements and established residential areas.

Eligible patients were >18-years of age, had been diagnosed with rifampicin or rifampicin and isoniazid resistance from sputa taken and tested in Cape Town between June 2010 and December 2012, and had received MDR-TB treatment at one of the four PHC facilities. Patients with previous MDR-TB treatment were excluded as their pathway to care may have been different. Patients with additional drug-resistance, in hospital or in prison, still smear-positive or with loss to follow-up at the time of the scheduled interview were excluded (Fig. 2).

Patients diagnosed at the selected facilities were identified from an electronic laboratory database; those diagnosed elsewhere, but on treatment at these facilities, were identified through facility registers and clinical records. As patients in each diagnostic algorithm became eligible, PHC facility nurses enquired whether they were willing to participate and provided their contact details to researchers for structured interviews. We purposively sampled a subset from the four facilities for qualitative interviews, stratifying participants by age, gender and MDR-TB risk profiles. PHC facility nurses asked selected patients if they were willing to participate in the second interview. Recruitment continued until an appropriate range of participants was interviewed and saturation was achieved in terms of key findings in both algorithms and no new themes appeared to emerge. We interviewed 16 patients diagnosed in the LPA-based algorithm between 2010 and 2012 and 10 patients diagnosed in the Xpert-based algorithm between 2011 and 2012.

### The research team

The research team comprised a senior social scientist with oversight for this qualitative study (NL), two social science field researchers, two professional nurses, a health sciences researcher (MvN), a PhD medical researcher who was the principal investigator (PN) and a clinician (EdT). None of the researchers were involved in the delivery of health services or care of patients.

### Data collection

As part of the broader PROVE IT study, professional nurses reviewed clinical records and completed a case record form (CRF) with demographic and clinical information. Three to six months after MDR-TB treatment had commenced, field researchers contacted eligible patients, obtained informed consent, interviewed patients and completed a structured questionnaire (Interview 1) detailing the care-seeking pathway, health-care visits and services received. Shortly thereafter, field researchers contacted the patients selected for this study, took informed consent again and conducted in-depth qualitative interviews in English or the patient’s mother tongue (Interview 2). We elected to use interviews to elicit patients’ first-hand experiences of their pathway to care. For infection control purposes, interviewers and patients wore N95-respirators and interviews were conducted in well-ventilated, private spaces at PHC facilities. Both the CRF and structured questionnaire were reviewed prior to interviews and the information was used to probe and clarify responses provided by the patients.

A semi-structured interview guide with open-ended questions was used. Patients were asked to provide a detailed account of their experiences from symptom onset to MDR-TB treatment initiation. Aspects of the care pathway explored in detail included patients’ recognition of their initial symptoms, their decisions around where they sought medical care, their experience of these health services and their MDR-TB diagnostic and treatment initiation process. All interviews were digitally recorded, translated into English where necessary and transcribed.

### Data analysis

Data analysis was undertaken by three of the authors (PN, MvN, NL). Each analyst read interview transcripts several times during the course of the study to familiarise themselves with their content. Analysts used open coding to independently identify key issues and themes in each stage of the care pathway with constant comparison within and between groups. We jointly recorded key events and issues for each patient on a treatment journey matrix based on data from the interview, supplemented by data from the case report forms and structured questionnaires. Consensus was reached through discussion. We used a combination of deductive (having explored specific aspects of the care pathway and the motivation behind patients’ actions) and inductive analysis, identifying common and divergent themes emerging from the data that were not specifically elicited [33].

### Ethics

The City Health Directorate and Western Cape Health Department granted permission to undertake the study. The Health Research Ethics Committee at Stellenbosch University (IRB0005239) (N10/09/308) and Ethics Advisory Group at The International Union Against Tuberculosis and Lung Disease (59/10) approved the study and provided a waiver of informed consent for the use of routine data. Written informed consent, in the patient’s preferred language, was obtained prior to completion of the structured questionnaire and qualitative interviews. Patients were provided with refreshments but were not reimbursed financially for interviews.

## Results

We interviewed 12 female (6 in each group) and 14 male participants ranging from 20 to 58 years in age. Thirteen patients had previously been treated for TB and 17 were co-infected with HIV.

We present key themes in four components of the care-pathway: symptom recognition, health-care access, testing for MDR-TB and MDR-TB treatment initiation. We have combined the description of patients’ experiences for both the LPA and Xpert-based algorithms and have highlighted similarities and differences. We identified both enablers and barriers to early MDR-TB diagnosis and treatment and summarise these in Table 1.

**Table 1:** Barriers and enablers in the pathway to early MDR-TB diagnosis and treatment initiation

	Enablers	Barriers
Symptom recognition	•Symptom recognition based on history of previous TB	•Failure to recognise TB symptoms
		•Minimisation or denial of symptoms
	•Social contact with TB/MDR-TB patients	•Lack of awareness that TB can recur
	•Awareness of increased risk of TB amongst HIV-infected patients	•Incorrectly ascribing symptoms to HIV or other medical condition
Accessing health-care	•Perceptions of good quality service	•Negative perceptions of the public sector (over-burdened; long waiting times; negative staff attitudes; lack of privacy)
	•Convenience of free, accessible local services.	
	•Familiarity with service	
	•Family support	•Fear of an HIV diagnosis
	•Responsiveness of provider at first health contact	•Social construct of “being a man”, not admitting illness (seen as weakness)
MDR-TB Testing	•Attendance at facilities geared towards TB (i.e. offering both TB diagnosis and treatment)	•Entry point to care through the private sector
	•Availability of Xpert MTB/RIF	•Accessing facilities providing TB diagnostic but not treatment services.
	•Screening of all presumptive TB cases for drug resistance	•Health providers failure to test for TB / MDR-TB at initial health contact
	•Patient’s agency in specifically requesting TB screening services that were not offered	•Health providers’ failure to follow diagnostic algorithms
	•Patient’s agency in pursuing diagnostic processes after initial negative tests	•Interruptions to the diagnostic process due to dissatisfaction with the service, work and family commitments
		•Lack of money for transport to return to facility
		•Insensitive tests that fail to diagnose TB
		•Patients diagnosed clinically or on chest x-ray and started on 1^st^-line TB treatment
		•Failure to respond early to clinical deterioration for patients on 1^st^-line TB treatment
Initiating MDR-TB Treatment	•Health provider scheduling early return visits for MDR-TB test results	•Patients failure to return for follow-up appointments
	•Patients returning for scheduled appointments	•Delays in recalling patients
	•Availability of decentralised MDR-TB treatment	•Results not being available at follow-up appointments
	•Perceptions that staff cared about their patient’s well-being	•Family commitments preventing a return to facilities
		•Cultural beliefs and seeking traditional healthcare (often in another province)

### Symptom recognition

Patients described their symptoms, what they attributed these to and what led them to seek care. Most described symptoms typically associated with TB such as cough, night sweats, loss of appetite, weight loss, fever, chest pain and general malaise. Some, particularly those with HIV, described symptoms related to their co-morbidity as their primary concern, for example painful feet for a patient on antiretroviral treatment.

#### The role of previous TB treatment or social contact with TB patients

Half the patients had previously been treated for TB and many recognised their symptoms as a recurrence. Several responded by quickly seeking help at a PHC facility as this patient explained: *“I had all the symptoms that I had the last time when I had TB. So I wanted them to check [for TB]” (Xpert-4)*. Having had TB before did not always mean that patients recognised recurring symptoms or sought timely help. One patient explained: “*I did not believe it could be TB again because I completed my treatment the first time” (Xpert-9).* Several patients reported that contact with someone on TB or MDR-TB treatment heightened awareness of their symptoms: *“I thought I had TB because I stayed with someone who had TB and he did not take his treatment” (LPA-15).* Another explained: *“I did not know much about MDR-TB. My [relative] had MDR-TB and told me to check if I don’t have it” (Xpert-7).*

#### The interaction with HIV

For some HIV-infected patients, awareness of their increased risk of TB motivated their response: *“I was coughing, my bones pained and I was losing weight…I thought I had TB …I went to the ARV doctor because I had an appointment … and told him how I feel. I asked him to send me for a TB test” (LPA-15).* However other HIV-infected patients incorrectly ascribed their symptoms to HIV and delayed seeking care*.* Fear of being diagnosed with HIV presented a barrier to accessing early care as one patient explained *“My mother said I must go to the clinic for a TB test. She was worried that I may have TB because my [relative] also had TB. I did not want to go …too scared that if I go for a TB test they will also test me for HIV” (LPA-2).*

#### Failure to recognise or acknowledge TB symptoms

Patients who did not recognise symptoms as TB–related, tended to minimise their significance. Several attributed symptoms to other factors, including smoking tobacco and the weather: *“I was having a terrible cough and I was sweating at night, but this did not ring an alarm for me, because I still thought this was just a fever and the change of season and that everything was going to be fine” (LPA-3).* Others mistook symptoms for “flu”, asthma or other medical conditions: *“My grandfather had TB in 2006. I knew the symptoms of TB are when a person is coughing and getting very thin…I did not think about TB… I thought it was swine flu…Everyone was talking about swine flu”* (*LPA-16).*

Some patient’s minimised their symptoms to avoid causing undue worry to their family. Family roles and responsibilities and the expectations that come with these contributed, as one patient reported: *“at the time when I started to feel sick I felt that I had to act a little bit strong, to not let the family know how weak I really felt. I must not let them down. Although I could feel some pain I felt I must be a man to face this disease” (Xpert-7).*

Patients tended to show considerable tolerance for their symptoms, referring to these as “just a cough” or “just losing weight”. A patient who completely dismissed his symptoms said: *“But at all these times I was not sick. It was just a cough, sweat at night and I felt that I was also losing weight, nothing else, not a day I ever felt like I was sick” (LPA-8).* Symptom tolerance or denial resulted in some patients presenting for care when they were extremely ill, having lost substantial weight or being too ill to walk.

There was wide variation in the time from symptom onset to seeking health-care. Some, especially those who recognised they may have TB, took early action. Others delayed seeking care, sometimes for several months, until their symptoms were much worse. The stigma of possibly having TB did not emerge as an issue influencing patients’ actions. The findings on symptom recognition were similar for patients in both the LPA and Xpert-based algorithms. It was interesting to note that none of the patients in the Xpert group reported a heightened awareness of TB or of the newly introduced test.

### Accessing health-care

Patients described when and where they first sought care and what motivated their choice of provider. Although the public sector was the most common entry-point to care, several (about one third) first visited the private sector.

#### Public sector as the entry-point to care

Convenience, accessibility, familiarity with the services and cost were the commonest reasons for patients choosing the public health sector. Patients accessed and moved between CHCs and clinics and experiences differed between these. Several patients who first attended a CHC were treated with antibiotics for an acute chest infection and were not initially screened for TB; in comparison, most patients attending clinics were initially screened for TB, although not necessarily for MDR-TB.

Previously treated patients tended to return to their local clinic for care due to familiarity and satisfaction with the previous service: *“I had TB before and knew the symptoms of TB. I decided to go to the clinic where I stay…I was very satisfied with the treatment that I got [previously]” (Xpert-7).* Several patients chose to go to the clinic or CHC providing their antiretroviral treatment and requested TB tests at scheduled appointments; others were screened during antiretroviral treatment re-initiation.

A few patients specifically chose a CHC, because of the services offered, including emergency services and the availability of chest x-rays. A patient who attended for these reasons commented: *“I was very worried because I could not walk. I thought something serious is wrong with me. My [friend] called the ambulance” (LPA-10).* Like other patients who had attended a CHC, this patient commented on the difficulties in accessing care: *“[the CHC] was very full. I don’t like [the CHC] and would not like to go there again… Lots of chaos. The nurses rushed me, they don’t give me time to talk and explain what’s wrong with me. This is not right for me. They don’t care about the patient. Once I was there and the nurses just went on tea, even if the very sick people are waiting on them. I told the nurse about a sick, old man and she said “he must just wait”. They just don’t care about the patients” (LPA-10).*

Although long waiting times were a common complaint, a few accepted this status quo: *“I waited for a long time before I was attended, but I understand that is the way it is. There are lots of people that need to be attended to everyday here at the clinic. But I knew that at the end of the day I was going to get assisted. I just told myself that… I am sick already and I need help and in order for me to get help I must be patient” (LPA-5).*

In contrast, some patients reported positive experiences: *“…I was sure I was going to stand in a long queue, but I explained to the TB nurse what was wrong with me and without me standing in a queue she told me to go to the TB room” (LPA-8).* Another explained *“I was expecting long queues and sitting for ages before getting help. I am not sure what the situation is at the other clinics, but …there was no queue and I got helped within 10 min…Staff in the TB room is very helpful and treats the patients with respect” (Xpert-9).*

#### Private sector as the entry-point to care

Several patients first visited the private sector, most commonly general practitioners (GP), due to perceptions of better services. They perceived the public sector to be over-burdened, with long waiting times, negative staff attitudes and a lack of privacy. Waiting times, in particular, were considered a barrier: *“I don’t like coming to the clinic when I’m not feeling well…You wait for a long time before they can attend to you…At the doctors room the treatment and waiting time was very reasonable. I only had to wait for 20 min. Here at the clinic, you wait…you can wait for 8 hours here” (LPA-4).*

Having financial resources enabled patients to visit a GP; however, in some cases, they did not return as recommended due to financial constraints. Reflecting on their management in the private sector, several patients felt that visiting a GP had been a waste of time and money. One patient had the following advice for those with TB symptoms: *“I can advise them all to go to the clinic…. There are much better options at the clinic than the private doctors…lots of test which can be taken…lots of tests which will find out what is wrong with you….For someone to get those things you have to be patient though” (LPA-6).*

Patients that first sought help at a pharmacy often underestimated the seriousness of their symptoms, assumed these were self-limiting and self-medicated with cough mixtures.

We found no significant differences between the LPA and Xpert-based algorithms in the point of first health-care access or in patients’ experiences. Despite perceptions of long waiting times, lack of privacy and poor staff attitudes, most patients reported that they would recommend that family and friends with TB symptoms go directly to PHC facilities, to reduce the cost and time to diagnosis.

### Testing for MDR-TB

Patients described how they came to be diagnosed with MDR-TB; the steps involved and the time it took. With the exception of two patients, most experienced lengthy delays due to providers not testing for TB or MDR-TB at initial visits, failure in the testing technology and patient-related delays, including interruptions to the diagnostic process and missed follow-up appointments.

#### Delays due to the first health provider not testing for TB

None of the patients who initially visited a pharmacy were referred for TB tests; a few subsequently visited a general practitioner, before eventually being investigated for TB at their local PHC facility. This contributed to MDR-TB diagnostic delays. In one extreme example, a patient with persistent cough described visits to the same pharmacy over a 6-month period: *“I went back again and again to the pharmacy and got different medication every time. I must have gone there five times” (LPA-13).*

Most patients who visited general practitioners were diagnosed with acute respiratory infections and treated with antibiotics, without TB investigations. Some had chest x-rays taken and a few had sputum tests. Some patients who did not respond to initial treatment had early referral to PHC facilities; others who could not afford to continue with private sector care attended the public sector when their health deteriorated. Several patients who visited general practitioners experienced lengthy delays as a result of numerous visits and courses of antibiotics, before seeking or being referred to a PHC facility for investigation.

In the public sector too, particularly at CHCs, some patients with a chronic cough were treated with several courses of antibiotics before a TB test was done. After an initial general practitioner visit one patient reported: “*I was at [the CHC] for 24 hours in December …they told me that I had infection in my lungs and gave me the drip and antibiotics…In the same month I didn’t feel so well so I went back… and they gave me the same drip and antibiotics” (LPA-16).*

**Fig. 3:**
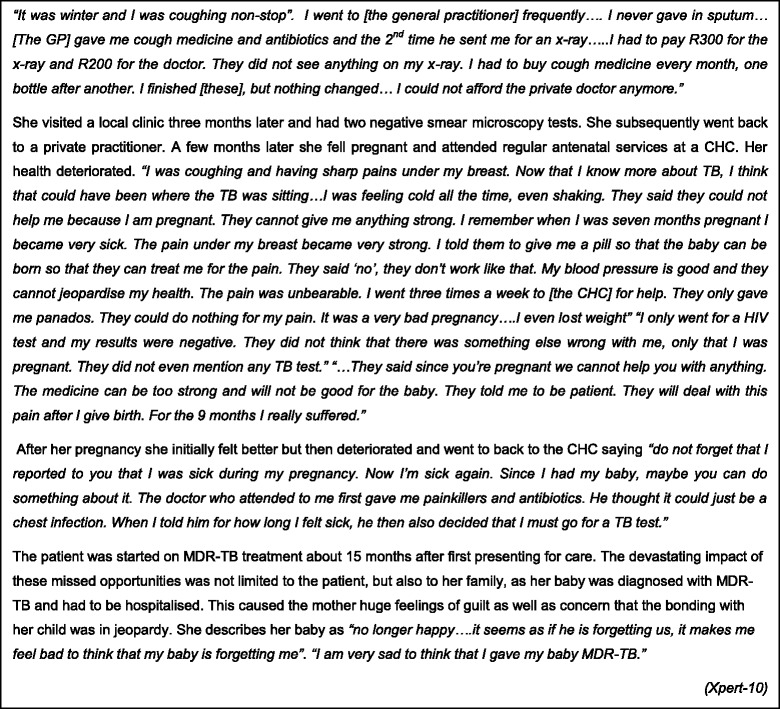
Delayed access to treatment - in their own words

In the most extreme example of missed diagnostic opportunities a young woman was started on MDR-TB treatment 15 months after first seeking health-care. Despite repeated visits to both the private and public sector, the patient was not adequately evaluated for TB, including during pregnancy. She was diagnosed several months after her baby’s birth with serious consequences including her baby contracting MDR-TB (see Fig. 3).

#### Delays due to the first health provider not testing for drug susceptibility

In the LPA-based algorithm, low MDR-TB risk patients did not have initial drug susceptibility tests (DST); this was done only when 1^st^-line TB treatment failed, resulting in substantial delays to MDR-TB treatment. In comparison, in the Xpert-based algorithm, *all* presumptive TB cases were required to have an Xpert test and were concurrently evaluated for tuberculosis and rifampicin resistance, theoretically reducing these delays. In both algorithms however, there were many instances where the correct tests were not initially done, contributing to diagnostic delays.

Patients who experienced delays commented on the distress they felt whilst on 1^st^-line TB treatment. For one patient, diagnosed on smear microscopy by a general practitioner and referred to a PHC facility, a series of health service failures contributed to delay. The patient was at high MDR-TB-risk and despite the clinician requesting an Xpert test, only smear microscopy was done and the patient was started on 1^st^-line TB treatment. Nurses did not respond to the patient’s complaint of worsening symptoms. After sputa were taken to evaluate response to treatment the patient reported: *“When the results came back they told me I do not take my tablets. I told them ‘but I take my pills every day’. They could not understand why my results were 3-plus positive. I told them ‘my [spouse] sees when I take my tablets in the morning’. This made me very troublesome… In all the time that I took the treatment I felt the same. The treatment did not help….I started to give up hope” (Xpert-6*). The patient started MDR-TB treatment almost 5-months after initial tests; following routine screening the patient’s child was also diagnosed with MDR-TB.

#### Delays due to initial negative or invalid tests

Patients in both algorithms were sometimes diagnosed on chest x-ray after negative initial smear or Xpert tests and commenced on 1^st^-line TB treatment as this patient explained: *“So finally after three days the results came back.… and a few days later they said I was negative, but I was still getting sicker.” (Xpert-8).* This patient persisted, seeking care from another facility: “*I went to [the CHC] again like someone who does not know what’s wrong with them. I told the doctor ‘I already gave sputum’ and I went for x-rays and that’s when she saw the x-ray and sent me straight here and they … put me on treatment”.* As per policy, this HIV-negative patient did not have an initial sputum culture test; this was only done two months after 1^st^-line TB treatment was started, contributing to a 4-month delay in MDR-TB treatment.

In comparison, a patient who was investigated for TB shortly after commencing antiretroviral treatment reported: “*I gave in the sputum and came back to the clinic after 3 days. The result was negative…I went again to the ARV clinic and they sent me for chest x-ray .My chest x-ray was also negative….I went back to work. [One day] I was preparing to go to work. My phone rang and it was the clinic that said I must come to them immediately” (Xpert-5).* The patient was diagnosed on a culture test, resulting in a 6-week delay to MDR-TB treatment.

#### Patients’ roles

Patient narratives presented several examples of their agency in persisting with the diagnostic process when symptoms did not resolve and in requesting tests that should have been offered but were not. One patient explained: *“I knew I was HIV positive, and that made me more worried when I felt sick. Even when my TB results were negative…I went again for a TB test” (LPA-14).* Whilst not playing a major role, patient factors contributed to delays during the diagnostic process due, for example, to work and family commitments and being unable to pay for transport to return to facilities.

#### A comparison between the LPA and Xpert-based Algorithms

There were many examples of remarkably similar patients’ experiences between the LPA and Xpert-based algorithms, with a 1–2 month delay between submitting the MDR-TB test sputum and treatment initiation. Delays overall were longer for patients in whom initial tests were negative with 1^st^-line TB treatment started on clinical or chest x-ray findings.

**Fig. 4:**
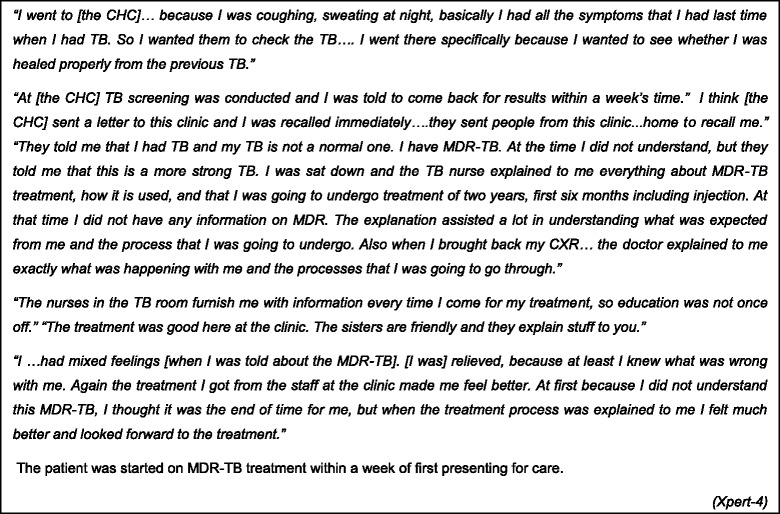
Expedited access to treatment - in their own words

With Xpert, there were two examples of rapid MDR-TB diagnosis where a test submitted at the first health contact diagnosed rifampicin resistance (see Fig. 4). A patient elucidated: *“At the clinic I was given the bottle to give sputum… After that I was given a follow-up date to come the following week for my results, and when I came back I was told that it looks like I have TB and that this TB is not like the first one…. I was told that I needed to see the doctor because I have a “big TB, MDR-TB” a more serious TB, not the normal one” (Xpert-2).*

### MDR-TB treatment initiation

Patients described their experiences of receiving test results and initiating treatment. We identified both health system and patient factors contributing to delays.

#### Health system factors contributing to delay

Problems were experienced with facilities failing to receive laboratory results, resulting in multiple return trips, as one patient explained: *“After returning for my results and waiting for a long time, I was told that I needed to come back again after two days. After another two days I was told my results were not received due to a broken fax machine. After this day I decided not to come back because I was waiting too long in the queue for my results and I was feeling better at this stage” (LPA-5).*

Once the PHC facility received the MDR-TB test result, there were delays in recalling patients, although from patient accounts, the reasons for this were not always clear. Several patients indicated that a health worker was able to contact them telephonically or visited their home. Some indicated that the PHC facility struggled to contact them initially, for instance, when messages were not relayed to them. Substantial delays occurred in recalling some patients. One patient who tested smear-negative in April and went to visit family in another province to recuperate reported: *“In mid-August the ..clinic phoned me to check when I will be back home. They did not say why. I only went back at the .end of August and was informed…that I have MDR-TB. This was another shock for me. I was very disturbed that the clinic [had] not told me this while I was [away]. I was living unaware of this very contagious disease…I think this was very irresponsible of the clinic. I told them… when they explained the seriousness of MDR-TB to me….I was a danger to my family and could have been on treatment 2-weeks earlier“ (Xpert-9).* In this extreme example, the patient was not aware that her results were in fact available three months prior to the patient being contacted.

#### Patient factors contributing to delay

Delays occurred when patients failed to return for scheduled appointments and had to be recalled. For a few, seeking traditional health-care at their family home in another province contributed to delays, as did family commitments. One patient explained: *“The day..I was told I have MDR-TB, my family phoned…with the news that my sister passed away. Everything then went crazy. All I could think about then was the fastest way to get [home.] I am the eldest son and must make all the preparation and decisions for the funeral. I left very early the next morning…not thinking about my MDR-TB treatment, maybe because my mind was very occupied with my family responsibilities and also because I did not feel that sick” (LPA-12).*

#### The impact of an MDR-TB diagnosis

Patients spoke at length about the range of emotions they experienced on receiving an MDR-TB diagnosis. Although devastating for most patients, the diagnosis was often accompanied by a sense of relief at finally knowing what was wrong with them. For many experiencing financial and other hardships, diagnostic delays exacerbated their difficulties. There were also feelings of guilt about infecting others, especially children: *“ it hurts me a lot, I don’t even want to go there, I am feeling very bad, very, very bad, because if this was detected earlier I was not going to go through some difficulties that I went through. You know… when I think that I even infected my child it makes me feel very bad. Because if this was detected early and [I was] started on the right treatment, maybe some of the problems would have been eliminated” (LPA-3).* Overall, six children in the households of the 26 patients interviewed were diagnosed with MDR-TB.

## Discussion

Limited data are available on the impact of new molecular diagnostic tests on MDR-TB patients. Two studies comparing conventional DST to LPA showed reductions in median treatment commencement times from 72 to 24 days [6] and 80 to 55 days [7] respectively. The PROVE-IT evaluation found a reduction from 43 days in the LPA-based algorithm to 17 days in the Xpert-based algorithm [8]. These findings however provide a limited view, excluding the period prior to MDR-TB tests being taken and do not explain, for example, why it takes 17 days to get a patient onto treatment despite a laboratory turn-around time of <1 day [8]. There is a paucity of literature detailing patients’ experiences of MDR-TB diagnosis and treatment initiation and this is one of the few studies to report specifically on this.

Our findings show that patients’ pathways to MDR-TB care varied: some were expedited, with treatment initiated within a week of first health provider contact, thus achieving the anticipated benefit of Xpert. However obstacles at all stages in the pathway delayed treatment for many patients in both the LPA and Xpert-based algorithms. Some of these delays could be considered as unavoidable (perhaps due to the complexity of the disease and the limitations of tests), but some, especially those related to health service failures, are clearly avoidable. We reflect on the implications of these findings for reducing delays in the pathway to MDR-TB treatment initiation.

### Symptom recognition

We found no differences in patients’ recognition of their symptoms in the LPA and Xpert-based algorithms. The media coverage during the launch of Xpert in South Africa did not appear to impact on patient’s health-seeking behaviour. Instead, factors that contributed to early symptom recognition and health-seeking in both algorithms included a previous history of TB, social contact with someone on treatment (both of which are unhelpful from a public health perspective) and awareness amongst some co-infected with HIV of their increased risk of TB. However, many patients deferred health-seeking for lengthy periods of time. As was found in other studies, some waited to see if symptoms like cough resolved spontaneously or improved with self-medication [11, 13, 14]; others deferred for fear of an HIV diagnosis [11–13] and patients who were uncertain of the cause of their illness delayed health-seeking or visited multiple providers [13–15]. The extent to which patients deferred health-seeking is difficult to ascertain bearing in mind the tendency to tolerate, underplay or deny TB symptoms. This is likely to be a significant issue in our setting, considering the chronic nature of TB and how ill many patients were at their first health contact.

Studies have reported equivocal findings on the role of TB knowledge in treatment delay [10,19, 20], with some finding an association between poor knowledge and delay [21–24] and others not [15, 25]. Our findings suggest that knowledge about symptoms and perceptions of risk influenced health-seeking. Raising awareness of TB symptoms and the need to seek early care may contribute to reducing patient delays. Perhaps the heightened awareness amongst those with previous TB presents an opportunity: these individuals could be targeted by education programmes to assume the role of “cough advocates” in their communities, with benefits for their own health too, as they are at higher risk of re-infection [26, 27]. The introduction of a new test like Xpert presents an opportunity to influence health-seeking behaviour through awareness campaigns that increase demand for the test.

#### Health-care access

About one third of the patients in our study first sought care in the private sector, due to perceptions of poor treatment in the public sector, particularly long waiting times and poor staff attitudes. Poor TB screening practices in the private sector contributed to delays for many patients. Other studies have also found that visits to private providers and facilities not equipped to diagnose and treat TB contributed to delays [13, 14, 19, 22, 28, 29]. Perceptions of poor public sector services are frequently cited as contributing to delay [11, 13–15] and may have contributed both to deferred health-seeking and to patient’s choice of the private sector in our study. Avoidance of free public sector services calls for serious reflection on how to improve service delivery. Improved TB screening practices in the private sector and early referral to the public sector are required to help reduce delays.

It is important to note that despite widespread negative perceptions of the public sector, several patients reported positive experiences at their first contact and almost all reported positive experiences once on treatment and indicated that they would recommend these services to family and friends. We do not feel that conducting the interviews at the health facilities influenced this sentiment as patients spoke candidly about negative experiences. Efforts to improve public health services could build on the positive sentiments expressed by patients.

#### Testing for MDR-TB and treatment initiation

Despite most patients, including those who were HIV-infected, presenting with symptoms typical of TB, one of the commonest factors contributing to delay in both diagnostic algorithms was health provider’s failure to test for TB at initial contact. This occurred most frequently when patients visited pharmacies, general practitioners and facilities not providing TB treatment. Large population-based surveys of health-seeking behaviour in other countries reported similar findings: the majority with chronic cough presented for care [23, 25, 30, 31] but only a small percentage were evaluated for TB [30, 31]. Efforts need to be made to increase the “index of suspicion” of TB amongst both private and public sector providers, especially those not providing TB treatment, to ensure appropriate testing.

An advantage of the Xpert-based algorithm is that by targeting all presumptive TB cases and not only those at high MDR-TB risk, it should diminish the problem of patients with primary MDR-TB being placed on 1^st^-line TB treatment and the ensuing suffering that patients described as their health deteriorated.

The potential of an Xpert-based algorithm to substantially reduce delay is highlighted by two examples of rapid initiation of MDR-TB treatment, within 6 and 8 days of the first health contact, respectively. Early access to treatment was enabled by the correct tests being requested which yielded a positive result, results being available when patients returned and decentralised treatment being available. In comparison, the earliest access for three patients in the LPA-based algorithm was within 31–38 days of the first health contact, reflecting the time taken for a culture and DST result with LPA.

However, more often than not, health system factors including failure to adhere to testing algorithms, problems with receiving the results, scheduling follow-up visits and recalling patients with positive results, all contributed to substantial delays. The introduction of rapid diagnostics therefore needs to be coupled with measures that address these structural barriers and minimize organizational delay [20, 32]. Patient-related delays (due to missed follow-up appointments, competing family demands and seeking traditional health-care) contributed to a lesser extent.

Our findings highlight not only the factors influencing the pathway to MDR-TB treatment, but also the impact on the lives of patients. Patients described how their physical and emotional suffering during the long and sometimes, tortuous pathway to treatment initiation compounded already difficult socio-economic and family circumstances. For some, the impact on their families was experienced as devastating, in particular, when children were infected. There was recognition (and anger) amongst some, that reducing diagnostic delays may have averted some of these infections.

#### Limitations and strengths

The study has limitations. It was undertaken in Cape Town which is urban, relatively well resourced and with decentralized MDR-TB care. Obstacles to care may be greater in poorly resourced areas, in rural settings and with centralized care. The study does not reflect the experience of the sickest MDR-TB patients (those in hospital or still smear positive), those not initiating MDR-TB treatment and those who were lost to follow-up, and may therefore present a more optimistic view of pathways to care. One of the major strengths of this study is that we were able to increase the validity of patient reports by triangulating data from interviews with clinical information and data from a structured questionnaire on health visits and services received. This also provided important context to patient’s narratives and deepened our understanding of pathways to care. Whilst the small sample included in the study may not have been statistically representative of all MDR-TB patients, we felt that we had captured an adequate range of experiences. Qualitative research, where patients are required to convey their experiences and the meaning they attribute to these [33] contributes important evidence on the impact of molecular diagnostic tests.

## Conclusion

The history of TB control efforts around the world has shown that having the right technology will not by itself resolve complex medical and public health challenges [34, 35]. We are likely to confront a range of barriers in making the most of new diagnostic technologies [36]. Whilst the introduction of Xpert clearly has the potential to reduce MDR-TB diagnostic and treatment delays, this alone does not suffice. Addressing patient delays in health-seeking is important. However, unless the structural barriers to care (correct evaluation at the first health contact, appropriate referral between sectors, developing patient-friendly health systems that are better organised to access results and commence treatment) are also addressed, the potential of rapid molecular diagnostic tests such as Xpert are unlikely to be fully realised. We hope that patients’ perspectives will be a call to action to address the obstacles identified in the pathway to MDR-TB treatment initiation.

## Abbreviations

CHC: Community health centre
CRF: Case record form
DST: Drug susceptibility test
GP: General practitioner
HIV: Human immunodeficiency virus
LPA: GenoType® MTBDR*plus* line probe assay
MDR-TB: Multidrug-resistant tuberculosis
MTB: Mycobacterium tuberculosis
PHC: Primary health-care
TB: Tuberculosis
Xpert: Xpert® MTB/RIF
XDR-TB: Extensively drug resistant tuberculosis
